# Long duration of immunity against a type 1 heterologous PRRS virus challenge in pigs immunised with a novel PRRS MLV vaccine: a randomised controlled study

**DOI:** 10.1186/s40813-018-0087-4

**Published:** 2018-05-16

**Authors:** Jeremy Kroll, Mike Piontkowski, Poul H. Rathkjen, Francois-Xavier Orveillon, Christian Kraft, Oliver G. Duran

**Affiliations:** 1 Boehringer Ingelheim Animal Health, 2412 South Loop Dr, Ames, IA 50010 USA; 2 Boehringer Ingelheim Animal Health, 2621 North Belt Highway, St. Joseph, MO 64506 USA; 3 Boehringer Ingelheim Vetmedica GmbH, Binger Straße 173, 55216 Ingelheim, Germany; 4 Boehringer Ingelheim Veterinary Research Center GmbH & Co. KG, Bemeroder Str. 31, 30559 Hannover, Germany

**Keywords:** Vaccine, Efficacy, PRRS, Immunity, Lung lesions, ADWG

## Abstract

**Background:**

Porcine reproductive and respiratory syndrome virus (PRRSV) is widespread in commercial pig farms worldwide, and has a significant cost to the swine industry. Herd owners need a vaccine that will confer long-lasting immunity to prevent PRRSV infection and transmission. The studies described here evaluated duration of immunity conferred by a European-derived PRRS (isolate 94,881) modified live virus (MLV) vaccine, Ingelvac PRRSFLEX® EU, at 20, 24, and 26 weeks post-vaccination. Primary endpoints were the assessment of gross and histological lung lesions and viral RNA load in lung tissue 10 days following heterologous PRRSV challenge. Secondary endpoints included clinical observations, average daily weight gain (ADWG) and viral RNA load in serum 10 days post-challenge. Three blinded, vaccination-challenge efficacy studies were performed using separate cohorts of pigs (*n* = 56 per study). Pigs received either Ingelvac PRRSFLEX® EU (Group 1) or placebo (Groups 2 and 3). Groups 1 and 2 were subsequently challenged with heterologous European PRRSV isolate 205,817 at 20, 24 or 26 weeks post-vaccination.

**Results:**

Mean gross lung lesion scores were significantly lower in Group 1 than in Group 2 at 24 and 26 weeks (*p* <  0.0001), but not at 20 weeks (*p* = 0.299). Significantly lower mean histological lung lesion scores were observed in Group 1 versus Group 2 at 20 (*p* = 0.0065), 24 (*p* <  0.0001) and 26 weeks (*p* <  0.0001). Mean viral RNA load in lung tissue was significantly lower in Group 1 than in Group 2 (*p* <  0.0001) at 20 (*p* <  0.0001), 24 (*p* <  0.0001) and 26 weeks (*p* <  0.0001). Cumulative viral RNA loads in serum during days 1–10 post-challenge were significantly lower in Group 1 than in Group 2 (*p* <  0.0001) in all studies. A significant increase in ADWG was observed in Group 1 compared with Group 2 at 20 weeks (*p* = 0.0027) and 24 weeks (*p* = 0.0004), but not at 26 weeks (*p* = 0.1041). There were no significant differences in clinical signs post-challenge in any study.

**Conclusion:**

These results suggest that Ingelvac PRRSFLEX® EU confers long-term immunity to European heterologous PRRSV, which is maintained up to 26 weeks after vaccination, corresponding to the expected lifespan of commercial pigs.

## Background

Porcine reproductive and respiratory syndrome (PRRS) is a viral infection of pigs, causing pneumonia, reproductive failure, and increased mortality in young animals [1]. The disease was first identified in North America in the late 1980s [2] and soon after in Europe [3], and has since spread to other regions worldwide [4]. The disease has profound economic consequences, causing significant losses to the worldwide swine industry, and an estimated annual loss of 1 billion US dollars in North America alone [5]. The extent of animal suffering, along with these economic losses have led to a significant drive to develop effective prevention, control and elimination strategies.

PRRS is caused by the PRRS virus (PRRSV), which is a member of the *Arteriviridae* family of the order *Nidovirales*. Like all members of this family, PRRSV has a single-stranded, positive-sense RNA genome. There are two major genetic lineages: Type 1, which was originally isolated in Europe in 1990 [6], and Type 2, which was first isolated in North America [7]. These two genotypes emerged at approximately the same time, but their nucleotide sequences differ by approximately 40% [8, 9]. Indeed, there is also significant genetic variation within each genotype [5, 10]. This significant heterogeneity both between and within PRRSV genotypes has hindered attempts to develop an effective vaccine against PRRS.

At present, two types of vaccine are commercially available: killed-virus (KV) vaccines and modified-live virus (MLV) vaccines [11, 12]. KV vaccines are largely ineffective, or have only limited efficacy [13–15] while MLV vaccines are generally regarded as the most effective [5], reducing reproductive and respiratory symptoms of PRRS disease, and improving weight gain in growing pigs [16].

Despite their promising efficacy, PRRS MLV vaccines are still associated with multiple problems. Commercially available PRRS MLV vaccines are based on either PRRSV Type 1 or 2 viral strains, which effectively induce immunity against genetically similar PRRSV strains from within the same genotype [11, 12]. However, the level of efficacy conferred against heterologous PRRSV isolates is less clear. Vaccine efficacy can suffer from both the immune evasion strategies of the virus and the antigenic heterogeneity of the field strains. It is not possible to forecast precisely the level of protection afforded by a given PRRSV vaccine strain against a heterologous one, but it is clear that partial heterologous protection can be obtained [17–19].

Commercially-reared pigs are typically slaughtered between 18 and 26 weeks of age [20]; therefore, an optimal vaccine should quickly elicit an effective immune response, and maintain duration of immunity for at least 26 weeks. The absence of clear correlates of protection means that further in vivo studies are necessary [21]. Current PRRS MLV vaccines elicit humoral and cell-mediated immunity; both become detectable by 2–3 weeks post-vaccination [22, 25]. However, whilst levels of PRRS-specific antibodies rapidly reach their peak of detection 4 weeks after vaccination or infection, the cell-mediated response is profoundly delayed, remaining at low levels for over 3 months before reaching its highest levels after 32 weeks post-vaccination [22]. As well as their efficacy shortcomings, there also remain concerns over the safety of PRRS MLV vaccines due to their potential to revert to virulence [23, 24]. A vaccine conferring rapid, long-lasting as well as effective immunity against a broad range of heterologous viral strains is very much needed [11, 23].

An ideal vaccine would achieve a long duration of immunity to reduce the risk of re-infection with PRRS during an animal’s lifetime. Simultaneously, it must effectively reduce clinical signs of PRRS disease, viraemia, viral RNA load in lung tissues and viral shedding, to help limit viral transmission. Such a vaccine would help limit both the acute economic losses caused by PRRS infection and animal suffering. Developing such a vaccine represents a significant challenge [23]. A new vaccine, PRRS 94881 (tradename: Ingelvac PRRSFLEX® EU), is derived from European PRRS isolate 94881, and has previously been shown to be clinically safe [25]. In order to determine whether PRRS 94881 MLV could provide long-term immunity to PRRSV challenge in 2-week old pigs, three vaccination-challenge studies following Good Clinical Practices (GCP) were carried out to evaluate the duration of immunity (DOI) at three different time points after vaccination. This paper details the results of these studies, which found that vaccinated pigs had reduced lung lesion scores, viraemia and viral RNA load in tissues at 20, 24 and 26 weeks.

## Methods

### Study designs

Three blinded, vaccination-challenge efficacy studies were performed using separate cohorts of pigs. Each study comprised three treatment arms. Group 1 (PRRS-vaccinated) received Ingelvac PRRSFLEX® EU (Boehringer Ingelheim Vetmedica, Inc., St. Joseph, MO, USA; Lot 390–005) followed by PRRSV challenge. Group 2 (challenge controls) received control product (CP; Boehringer Ingelheim Vetmedica, Inc.; Lot N240–191-062409) followed by PRRSV challenge. Group 3 (negative controls) received CP, but no PRRSV challenge. Ingelvac PRRSFLEX® EU vaccine and CP were administered to 14–17 day-old pigs on Day 0, and then Group 1 and Group 2 were challenged with heterologous European PRRSV isolate 205,817 (Boehringer Ingelheim Vetmedica, Inc.) at 20, 24 or 26 weeks after vaccination. This day was denoted day post-challenge (DPC) 0. All studies were carried out following Good Clinical Practice (GCP) guidelines.

### Randomisation and blinding

Animals were randomised to one of three groups by the study investigator or a designee prior to study commencement. The randomisation sequence was created using Excel 2003 (Microsoft, Redmond, WA, US) with a 1:1 allocation using random block sizes of 0 and 1. Both the Ingelvac PRRSFLEX® EU and CP were administered by individuals not collecting study data in order to maintain study blinding. Both the study investigator and all laboratory personnel were blinded to treatment assignment.

### Animals

These three studies were each conducted with 56 commercial crossbred female or castrated male pigs (Prairie View Farms, N5627 Hwy DD, Burlington, WI 53105, USA). The animals were healthy, aged between 14 and 17 days, weighed between 2.7–6.3 kg and were PRRSV-negative on Day 0. All pigs were housed at Veterinary Resources, Inc. (VRI) in Cambridge, IA, USA, for the duration of the study. Pigs were housed in multiple pens (each containing 11–12 pigs) per room. Vaccinated (Group 1) and control (Groups 2 and 3) animals were housed in uniform but separate rooms to prevent PRRSV cross-contamination between groups. Feeds provided were appropriate for the size, age and condition of pigs according to acceptable animal husbandry practices for the region. A minimum of 20 pigs were included in Groups 1 and 2, and 12 pigs were included in Group 3.

### Vaccines and challenge material

Both Ingelvac PRRSFLEX® EU vaccine and CP were reconstituted with phosphate-buffered saline. Ingelvac PRRSFLEX® EU was reconstituted and administered to Group 1 animals per manufacturer’s instructions [26]. Ingelvac PRRSFLEX® EU vaccine and CP were administered intramuscularly (IM) as a 1.0 mL dose to the right neck region of Group 1 and 2 pigs, respectively. Challenge material was PRRSV isolate 205,817, a heterologous Type 1 isolate with 88.3% homology to Ingelvac PRRSFLEX® EU (based on ORF5 sequence). The challenge strain was originally isolated from a herd with a severe outbreak of PRRS causing abortions in sows and respiratory disease in fattening pigs. Challenge material had a mean viral titre of 1 × 10^6.27^ TCID_50_/3 mL dose administered 1 mL per each nostril (2 mL in total) and 1 mL intramuscularly. The CP was a lyophilised placebo product that contained an inert material comprising the vaccine vehicle without Ingelvac PRRSFLEX® EU.

### Variables for efficacy and safety assessments

The primary efficacy outcome variable for the three studies was lung pathology (gross and histological lesions) at 20, 24 or 26 weeks after PRRS 94881 MLV vaccination. The DOI at 20, 24 and 26 weeks post-vaccination was achieved if Group 1 had statistically significant decreased lung pathology (gross or histological) post-challenge compared with Group 2 at the same time point. Secondary efficacy outcomes included post-challenge viraemia (in both lung and serum), average daily weight gain (ADWG), post-vaccination safety assessments and post-challenge clinical assessments of disease.

### Sample analysis and outcome measurements

#### Gross and histological lung lesions

Gross pathology was determined following examination of lung tissue following necropsy on DPC 10. A percentage of affected lung tissue was recorded for each lung lobe and the total percentage was subsequently calculated based on the weighing formula recommended in the draft monograph ‘Porcine enzootic Pneumonia Vaccine (inactivated)’ (PA/PH, Exp 15 V/T[07]2 ANP). To determine histological pathology, a single slide containing seven sections (one each for all seven lung lobes) was created for each pig. Slides were examined for pneumocytic hypertrophy and hyperplasia, septal infiltration with mononuclear cells, necrotic debris, intra-alveolar accumulation of inflammatory cells, and perivascular accumulation of inflammatory cells. For each histological parameter (except necrotic debris), samples were scored either 0 (not present: no detectable lesions present within an area of view), 1 (mild lesions: few positive cells [1–5 cells/area] present within an area of view), 2 (moderate lesions: multiple positive cells [> 5 cells/area] within an area of view) or 3 (severe lesions: multiple positive cells [> 5 cells/area] at multiple locations within an area of view). Necrotic debris was scored either 0 (not present) or 1 (yes present).

#### Serum PRRSV quantitative polymerase chain reaction

Two to 5 mL venous whole blood samples were collected from all pigs on Day 0 to confirm the PRRSV-negative status. Blood samples were also taken on DPC 0, 3, 7, 9 and 10 in all three studies. Reverse transcription and quantitative PCR (qPCR) were performed [27] on serum samples to determine serum PRRSV RNA levels; results were reported as genome equivalent/mL (log_10_ GE/mL).

#### Lung PRRSV qPCR

Tissue samples from left and right lung lobes were homogenised, and qPCR following reverse transcription was performed (BioScreen GmbH, Hannover, Germany) to determine lung PRRSV RNA levels [27]; results were reported as Log_10_ GE/mL.

#### Average daily weight gain

Body weights were recorded on Day 0, DPC 0 and DPC 9 in all studies, and individual daily weight gains were calculated between DPC 0–9.

#### Post-vaccination and post-challenge clinical safety observations

Post-vaccination clinical safety assessments were performed daily on Days − 1 to 21, then three times per week thereafter until DPC − 2 in all studies. These clinical assessments were recorded as either ‘normal’ or ‘abnormal’. Pigs were observed for clinical signs of disease from DPC − 1 to 10 in all three studies. Clinical parameters included respiratory symptoms, behaviour and cough, and were scored 0–3 based on severity of symptoms (Respiration: 0 = normal respiration; 1 = panting/rapid respiration; 2 = dyspnoea; 3 = dead. Behaviour: 0 = normal; 1 = mild to moderate lethargy; 2 = severely lethargic or recumbent; 3 = dead. Cough: 0 = no coughing; 1 = soft or intermittent cough; 2 = harsh or severe, repetitive cough; 3 = dead).

### Statistical methods

For each study, pigs were randomly assigned to one of three groups. Data were summarised using descriptive statistics with a 95% confidence interval (CI), and analysed assuming a completely random design structure. All tests on differences between Groups 1 and 2 were designed as two-sided tests using an alpha value of 5% (*p*-value < 0.05 for indicating statistical significance). Differences between treatment groups in each study were tested using analysis of variance in case of quantitative variables with normally distributed data (ADWG) and Wilcoxon Mann-Whitney tests for scores and other variables with not normally distributed data. Statistical analyses were performed using SAS software release 8.2 (SAS 2001, SAS Institute Inc., Cary, North Carolina, USA). No statistical analyses were performed on Group 3 pigs.

## Results

### Animals

In total, 56 animals were included per study, and assigned to one of three groups. For each study, Groups 1 and 2 comprised 22 animals each, and Group 3 comprised 12 animals. In the 20-week study, two pigs assigned to Group 2 died. In the 24-week study, one Group 1 pig and three Group 2 pigs died, and in the 26-week study, one Group 1 pig and two Group 2 pigs died. All of these animals died pre-challenge, and were not included in the analyses presented here.

### Lung lesion scores

The mean gross lung lesion scores for Group 1 pigs following challenges at 20, 24 and 26 weeks were 0.156%, 0.084% and 1.099%, respectively, whilst those for Group 2 pigs were 0.26%, 3.38% and 15.84%, respectively (Table 1). The differences between the two groups were significant after challenges at 24 and 26 weeks, but not after 20 weeks (Table 1).

**Table 1:** Gross lung lesion scores at necropsy

	Gross lung lesion score (%)					
Study	Group	n	Mean	95% CI	Median (IQR)	*p*-value*
20-week	1	22	0.156	0.00–0.16	0.000 (0.160)	0.2989
	2	20	0.261	0.00–0.16	0.060 (0.185)	
24-week	1	20	0.084	0.00–0.10	0.000 (0.130)	< 0.0001
	2	19	3.378	0.61–4.74	2.050 (4.59)	
26-week	1	21	1.099	0.05–0.55	0.060 (0.400)	< 0.0001
	2	19	15.842	2.69–22.65	13.80 (20.85)	

*CI* distribution free confidence interval of the median, *IQR* interquartile range, *MLV* modified live virus, *n* number of pigs included in analysis, *PRRS* porcine reproductive and respiratory syndrome

Group 1 = PRRS 94881 MLV vaccinated; Group 2 = challenge controls

^*^p-values were calculated using the Wilcoxon-Mann-Whitney test

Group 1 pigs exhibited significantly lower mean histological lung lesion scores than Group 2 pigs after challenges at 20, 24 and 26 weeks (Table 2).

**Table 2:** Mean histological lung lesion scores at necropsy

	Histological lung lesion score (mean)					
Study	Group	n	Mean	95% CI	Median (IQR)	*p*-value^*^
20-week	1	22	13.0	4.0–18.0	9.5 (14.0)	0.0065
	2	20	24.7	16.0–35.0	23.0 (20.5)	
24-week	1	20	7.3	2.0–10.0	7.0 (8.5)	< 0.0001
	2	19	20.6	12.0–28.0	19.0 (16.0)	
26-week	1	21	6.6	3.0–8.0	6.0 (5.0)	< 0.0001
	2	20	20.2	15.0–23.0	19.5 (10.0)	

*CI* distribution free confidence interval of the median, *IQR* interquartile range, *MLV* modified live virus, *n* number of pigs included in analysis, *PRRS* porcine reproductive and respiratory syndrome

Group 1 = PRRS 94881 MLV vaccinated; Group 2 = challenge controls

^*^p-values were calculated using the Wilcoxon-Mann-Whitney test

### Viral RNA load in serum

PRRSV RNA was not detected in the serum of any pigs on Day 0, meeting the inclusion criteria for the study. Also, no PRRSV RNA was detected in any group on the day of challenge (DPC0), meaning that the vaccine virus was cleared at this point in time.

Group 1 pigs in all three studies exhibited significantly lower serum PRRSV RNA levels than Group 2 pigs at both DPC 7 and DPC 10 (Table 3). During DPC 1–10, the mean area under the curve (AUC) values for Group 1 pigs were 14.3, 12.9 and 17.6 log_10_ GE/mL, and 37.4, 35.3 and 44.8 log_10_ GE/mL for Group 2 pigs in the 20, 24 and 26-week studies, respectively (Table 4).

**Table 3:** PRRSV-viral RNA load in serum

		PRSSV-viral RNA load in serum (log_10_ GE/mL)					
Study	DPC	Group	n	Mean	95% CI	Median (IQR)	*p*-value^*^
20-week	0	1	22	0.00	0.00–0.00	0.00 (0.00)	1.0000
		2	20	0.00	0.00–0.00	0.00 (0.00)	
	3	1	22	3.84	3.00–4.29	3.79 (1.29)	< 0.0001
		2	20	5.30	5.06–5.52	5.34 (0.54)	
	7	1	22	0.14	0.00–0.00	0.00 (0.00)	< 0.0001
		2	20	4.00	3.44–4.59	3.95 (1.26)	
	10	1	22	0.27	0.00–0.00	0.00 (0.00)	< 0.0001
		2	20	3.23	3.00–3.37	3.00 (0.51)	
24-week	0	1	20	0.00	0.00–0.00	0.00 (0.00)	1.0000
		2	19	0.00	0.00–0.00	0.00 (0.00)	
	3	1	20	3.39	3.00–4.04	3.78 (1.13)	< 0.0001
		2	19	5.06	4.85–5.36	5.13 (0.56)	
	7	1	19	0.32	0.00–0.00	0.00 (0.00)	< 0.0001
		2	19	3.85	3.00–4.53	3.87 (1.53)	
	10	1	20	0.00	0.00–0.00	0.00 (0.00)	< 0.0001
		2	19	2.75	3.00–3.37	3.00 (0.37)	
26-week	0	1	21	0.00	0.00–0.00	0.00 (0.00)	1.0000
		2	20	0.00	0.00–0.00	0.00 (0.00)	
	3	1	21	4.42	3.93–5.28	4.44 (1.51)	< 0.0001
		2	20	5.81	5.75–6.00	5.88 (0.32)	
	7	1	21	0.61	0.00–0.00	0.00 (0.00)	< 0.0001
		2	20	5.30	4.86–5.69	5.30 (1.08)	
	10	1	21	0.00	0.00–0.00	0.00 (0.00)	< 0.0001
		2	20	3.97	3.71–4.42	4.24 (1.18)	

*CI* distribution free confidence interval of the median, *DPC* day post-challenge, *GE* genome equivalent, *IQR* interquartile range, *MLV* modified live virus, *n* number of pigs included in analysis, *PRRS* porcine reproductive and respiratory syndrome, *PRRSV* PRRS virus

Group 1 = PRRS 94881 MLV vaccinated; Group 2 = challenge controls

^*^p-values were calculated using the Wilcoxon-Mann-Whitney test

**Table 4:** PRRSV-viral RNA cumulative loads in serum

			AUC values (log_10_ GE/mL)				
Study	DPC	Group	n	Mean	95% CI	Median (IQR)	*p*-value^*^
20-week	0–10	1	22	14.33	10.50–17.80	13.32 (7.3)	< 0.0001
		2	20	37.40	35.02–39.37	37.44 (4.54)	
	3–10	1	22	8.57	6.00–10.46	7.61 (4.46)	< 0.0001
		2	20	29.44	27.71–31.08	29.74 (3.76)	
24-week	0–10	1	19	12.87	10.50–15.16	13.23 (4.66)	< 0.0001
		2	19	35.31	32.78–38.22	36.07 (5.68)	
	3–10	1	19	7.83	6.00–8.86	7.56 (2.86)	< 0.0001
		2	19	27.72	25.16–30.72	28.29 (5.70)	
26-week	0–10	1	21	17.61	13.76–19.53	15.54 (5.95)	< 0.0001
		2	20	44.84	43.23–48.03	44.77 (6.24)	
	3–10	1	21	10.97	7.86–11.16	8.88 (3.40)	< 0.0001
		2	20	36.12	34.60–38.53	36.43 (5.23)	

*AUC* area under the concentration-time curve, *CI* distribution free confidence interval of the median, *DPC* day post-challenge, *GE* genome equivalent, *IQR* interquartile range, *MLV* modified live virus, *n* number of pigs included in analysis, *PRRS* porcine reproductive and respiratory syndrome, *PRRSV* PRRS virus

Group 1 = PRRS 94881 MLV vaccinated; Group 2 = challenge controls

^*^p-values were calculated using the Wilcoxon-Mann-Whitney test

AUC values during DPC 3–10 were also significantly lower for Group 1 than Group 2 pigs in all three studies. Group 1 pigs exhibited mean AUC values of 8.57, 7.83 and 11.0 log_10_ GE/mL compared with 29.4, 27.7 and 36.1 log_10_ GE/mL for Group 2 pigs in the 20, 24 and 26-week studies, respectively (Table 4).

### Viral RNA load in lung tissues at necropsy

Lung PRRSV RNA levels at necropsy for Group 1 pigs were significantly lower than for Group 2 pigs in all three studies (*p* <  0.0001; Table 5).

**Table 5:** PRRSV-viral RNA load for lung tissues at necropsy

	PRRSV-viral RNA load in lung tissues at necropsy (mean log_10_ GE/mL)					
Study	Group	n	Mean	95% CI	Median (IQR)	*p*-value^*^
20-week	1	22	4.26	3.70–5.18	4.20 (1.51)	< 0.0001
	2	20	6.31	5.94–6.73	6.51 (0.86)	
24-week	1	20	2.36	1.50–4.17	1.50 (3.42)	< 0.0001
	2	19	5.33	4.85–5.97	5.34 (1.23)	
26-week	1	21	3.36	1.50–5.21	3.69 (3.18)	< 0.0001
	2	20	6.22	5.62–6.68	6.25 (1.26)	

*CI* distribution free confidence interval of the median, *GE* genome equivalent, *IQR* interquartile range, *MLV* modified live virus, *n* number of pigs included in analysis, *PRRS* porcine reproductive and respiratory syndrome, *PRRSV* PRRS virus

Group 1 = PRRS 94881 MLV vaccinated; Group 2 = challenge controls

^*^p-values were calculated using the Wilcoxon-Mann-Whitney test

### Average daily weight gain

At the time of challenge, the mean weight difference between respective groups was insignificant. A significant increase in ADWG was observed in Group 1 pigs compared with Group 2 pigs in the 9 days following challenge at 20 (*p* = 0.0027) and 24 (*p* = 0.0004) weeks, but not at 26 weeks (*p* = 0.1041) (Table 6). Mean ADWG in Group 3 pigs was 1.016, 0.771 and 0.5 kg/day in the 9 days following challenge at 20, 24 and 26 weeks, respectively. This was greater than the ADWG of both Group 1 and 2 pigs in all three studies.

**Table 6:** Mean group ADWG during DPC 0–9

Mean ADWG (kg/day)					
Study	Group	n	Mean (SD)	Median	*p*-value^*^
20-week	1	22	0.740 (0.333)	0.739	0.0027
	2	20	0.155 (0.783)	0.339	
24-week	1	20	0.737 (0.295)	0.683	0.0004
	2	19	0.068 (0.705)	0.189	
26-week	1	21	0.412 (0.317)	0.411	0.1041
	2	20	0.235 (0.365)	0.289	

*ADWG* average daily weight gain, *MLV* modified live virus, *n* number of pigs included in analysis, *PRRS* porcine reproductive and respiratory syndrome, *SD* standard deviation

Group 1 = PRRS 94881 MLV vaccinated; Group 2 = challenge controls

^*^p-values were calculated using the analysis of variance (t-test)

### Clinical signs post-challenge

For all three studies, no difference was observed between Group 1 and Group 2 for each of the clinical signs considered: abnormal respiration, abnormal behaviour and coughing. No pigs in either Group 1 or Group 2 exhibited any coughing following challenge at 20, 24 or 26 weeks. No abnormal respiration, behaviour or coughing was observed in any pigs challenged at 20 weeks.

### Adverse events

No adverse events attributed to the test product (Ingelvac PRRSFLEX® EU) was noted in any of the three studies. Also, no deaths were attributed to either control product or Ingelvac PRRSFLEX® EU.

## Discussion

These three GCP laboratory efficacy studies were performed in seronegative 2–week old pigs. Each study contained three groups; Group 1 was vaccinated on Day 0 with Ingelvac PRRSFLEX® EU, and Groups 2 and 3 were vaccinated with a placebo vaccine. This study aimed to evaluate DOI at 20, 24 and 26 weeks following vaccination. After 20, 24 or 26 weeks, Groups 1 and 2 were challenged with a heterologous European PRRS viral isolate 205,817, which shared 88.3% sequence homology at the GP5 gene with Ingelvac PRRSFLEX® EU. The GP5 gene encodes the major envelope protein of PRRSV, and carries the major neutralising epitope of PRRSV [28]. Groups 1 and 2 were subsequently evaluated for lung pathology, serum and lung viremia, and observed for clinical signs of disease. No infections were observed in Group 3 pigs in any study, and there were no signs of co-infection. Therefore, the results presented here were attributed to the effects of PRRS 94881 MLV vaccine.

PRRS respiratory infection models using EU type 1 PRRSV are rare due to them being difficult to develop. In the current study, we have modelled a novel PRRSV type I isolate 205,817 that can consistently infect and cause severe PRRS respiratory lesions at different ages of a growing and fattening pig; however, the amount of expected clinical signs and respiratory disease is reduced the older the pig gets.

Reduction in gross and histological lung lesion scores was the primary efficacy endpoint of three studies. Lung lesion development is one of the hallmarks of PRRSV in growing pigs [29], and can be considered the source for all subsequent manifestations of secondary PRRS disease characteristics, including clinical signs, pyrexia, decreased ADWG and secondary infection with other pathogens. Therefore, these are the most clinically relevant and convincing parameters for measurement of PRRS vaccine efficacy. Macroscopic gross lung lesions are usually mild in adult animals, so slides were examined for multiple microscopic lung lesions typically observed following PRRSV infection [30]. The results of these three studies showed significant improvements in both gross and histological lung lesion scores among Group 1 compared with Group 2 pigs at 24 and 26 weeks after vaccination. Additionally, a significant reduction in histological lung lesions was seen at 20 weeks in Group 1 pigs. In the 20-week study, the lung lesion score of Group 1 animals was in a similar range to that of Group 1 animals in the two adjacent studies; however, significance could not be reached due to the fact that lung lesions in Group 2 pigs were low as well. These findings suggest that Ingelvac PRRSFLEX® EU vaccine is highly effective in providing long-term (up to 26 weeks) immunity against a virulent challenge with PRRS. Some histological lung lesions were observed in negative control pigs, despite their confirmed absence of PRRSV RNA in serum at all time points throughout the study. Pigs housed under normal swine husbandry conditions for extended periods of time can develop minor lung lesions that are inconsequential, and not related to specific pathogens [31].

Post-challenge viremia was selected as the most important secondary efficacy parameter because it represents the level of viral replication occurring within the host animal upon exposure. Furthermore, pathogenic and humoral immune responses to PRRSV are related to viral loads in acute infection [32]. Therefore, a significant reduction in PRRS viral load following vaccination would indicate that the vaccine could efficiently limit PRRSV pathogenesis in the host. Significant reductions in post-challenge viremia were observed in Group 1 pigs compared with Group 2 pigs 7 and 10 days post-challenge at 20–26 weeks. Cumulative exposure (AUC) from Days 3–10 post-challenge was also decreased in these pigs. Both findings indicate that Ingelvac PRRSFLEX® EU is efficient in limiting PRRSV viremia following virulent challenge, and confers a duration of immunity up to 26 weeks. Furthermore, a significant reduction in PRRSV-viral RNA load in lung tissue was observed in Group 1 pigs compared with Group 2 pigs at 20, 24 and 26 weeks. Viral RNA load in lung tissue is associated with viral replication and persistence in the host [33]. The results of these studies showed that Ingelvac PRRSFLEX® EU can effectively reduce PRRS-viral RNA load in lung tissue.

PRRSV infection contributes to a reduction in daily weight gain in young pigs [34]. In the 9 days following PRRSV challenge at 20 and 24 weeks, the ADWG of the Group 1 pigs was significantly higher than that of Group 2 pigs, whose ADWG was lower than that of Group 3 pigs. Following challenge at 26 weeks, ADWG in Group 1 pigs was increased compared with Group 2, but this increase did not reach statistical significance. These results indicate that Ingelvac PRRSFLEX® EU is effective at diminishing weight gain reduction caused by PRRS, possibly representing substantial economic importance. A recently published field study also supports this conclusion. This study found that vaccinating 4-week old piglets with Ingelvac PRRSFLEX® EU improved weight gain and reduced clinical signs during the growing period, even when the piglets are infected shortly after vaccination [35]. However, the current study is limited by the lack of statistical power due to the small sample size and findings need to be confirmed in larger studies. Also, this study used young pigs that were negative for PRRSV RNA; further studies are therefore required to determine whether these results apply to pigs of different ages and immune status.

PRRS MLV vaccines are considered the most effective PRRS vaccines available [5, 12]. The results of the previous studies provide evidence that Ingelvac PRRSFLEX® EU effectively confers immunity to PRRSV, which is maintained for up to 26 weeks. Since commercially-reared animals are typically slaughtered between 18 and 26 weeks of age [20], this long DOI conferred by Ingelvac PRRSFLEX® EU may help reduce the chance of PRRSV-related disease for the whole lifespan of vaccinated animals.

## Conclusions

The three studies described here show that Ingelvac PRRSFLEX® EU was highly effective at conferring long-term immunity (up to 26 weeks) against virulent challenge with a heterologous European isolate of PRRSV in young pigs. After challenge at 24 and 26 weeks, gross and histological lung lesion scores, post-challenge viremia and viral RNA load in lung tissue, and ADWG were improved in PRRS-vaccinated pigs compared with challenge controls, with most measures reaching statistical significance. These results show that protection against heterologous PRRSV is achieved with the Ingelvac PRRSFLEX® EU, and can be maintained for the expected lifespan of young pigs, with beneficial effects on animal health and production.

## Abbreviations

ADWG: average daily weight gain
AUC: area under the concentration-time curve
CI: confidence interval
CP: control product
DOI: duration of immunity
DPC: day post-challenge
GCP: good clinical practices
GE: genome equivalent
IM: intramuscular
IQR: interquartile range
KV: killed virus
MLV: modified live virus
PRRS: porcine reproductive and respiratory syndrome
PRRSV: porcine reproductive and respiratory syndrome virus
qPCR: quantitative polymerase chain reaction
TCID: tissue culture infective dose
VRI: Veterinary Resources, Inc.
